# Solid waste management in primary healthcare centers: application of a facilitation tool**^1^**

**DOI:** 10.1590/1518-8345.0646.2768

**Published:** 2016-08-18

**Authors:** Ana Maria Maniero Moreira, Wanda Maria Risso Günther

**Affiliations:** 2 Doctoral Student, Faculdade de Saúde Pública, Universidade de São Paulo, São Paulo, SP, Brazil.; 3 PhD, Associate Professor, Faculdade de Saúde Pública, Universidade de São Paulo, São Paulo, SP, Brazil.

**Keywords:** Medical Waste, Hazardous Waste, Waste Management, Indicators, Health Center

## Abstract

**Objectives:**

to propose a tool to facilitate diagnosis, formulation and evaluation of the Waste Management Plan in Primary Healthcare Centers and to present the results of the application in four selected units.

**Method:**

descriptive research, covering the stages of formulation /application of the proposed instrument and the evaluation of waste management performance at the units.

**Results:**

the tool consists in five forms; specific indicators of waste generation for outpatients healthcare units were proposed, and performance indicators that give scores for compliance with current legislation. In the studied units it is generated common waste (52-60%), infectious-sharps (31-42%) and recyclable (5-17%). The average rates of generation are: 0,09kg of total waste/outpatient assistance and 0,09kg of infectious-sharps waste/outpatient procedure. The compliance with regulations, initially 26-30%, then reached 30-38% a year later.

**Conclusion:**

the tool showed to be easy to use, bypassing the existence of a complex range of existing regulatory requirements, allowed to identify non-conformities, pointed out corrective measures and evaluated the performance of waste management. In this sense, it contributes to decision making and management practices relating to waste, tasks usually assigned to nurses. It is recommended that the tool be applied in similar healthcare units for comparative studies, and implementation of necessary adaptations for other medical services.

## Introduction

Services provided in healthcare facilities generate considerable amount of solid waste denominated as a whole, Healthcare Waste (HW). Much of this waste (75-90%) is considered similar to those generated in households (recyclable or not)^1^, while the rest, due to their hazardous characteristics (pathogenicity, toxicity and radioactivity) require different processes for management and treatment before disposal into the environment^1^.

The management of HW involves planning, implementation and monitoring of actions that aim to prevent exposure, ensure the safety of users and professionals involved, prevent the occurrence of environmental impacts while minimizing the generation of waste^1^ ^-^ ^2^.

Although the management of HW is routinely practiced by healthcare facilities, studies in developing countries^3^ ^-^ ^11^ indicate frequent inadequacies, such as inefficient management; failures in segregation and handling; lack of training and awareness of risks, insufficient human and economic resources for the right management; adoption of inadequate treatment techniques; lack of control over the endpoint and even shortcomings or absence of specific regulations.

In Brazil, in the last two decades, the legal and regulatory framework geared to HW^*^ suffered progressive updates, involving the ministries of Health, Environment and Labor, ending with the establishment of the National Policy of Solid Waste in 2010. All these Brazilian regulations and directives, which are aligned and complementary, provide that any healthcare facility in the country, regardless of size and complexity of the service, is responsible for managing its waste, and must prepare, implement and monitor its Healthcare Waste Management Plan (HWMP).

HWMP is the document that describes all internal and external steps for the management of waste in healthcare services, in order to prevent occupational accidents, to avoid environmental impacts and to protect the public health^3^ ^,^ ^12^. It could therefore go beyond a simple mandatory document and become an important supporting tool management. However, it has been widely assumed that, in spite of it being a legal requirement, the implementation of HWMP has not been a reality in the country^3^ ^-^ ^5^.

The literature indicates that the simple formulation of the HWMP, involving multiple aspects such as sanitary, environmental, health, and safety of the workers, has been a major challenge for healthcare institutions. Factors such as the lack of economic resources for the purchase of materials or equipment needed and the shortage of human resources also hamper the subsequent stages of implementation and monitoring plan.

In 2005, a study covering 21 hospitals and 48 outpatient public units of the State of Rio Grande do Sul, Brazil^5^, found that 28,6% (hospitals) and 4,2% (outpatients units) had deployed a HWMP, and only 33,3% and 10,4% respectively had developed employee training programs. Five years later, a new assessment carried out in nine Primary Healthcare Centers (PHC), in the Brazilian state of Goiás^3^ showed that none had HWMP or even a technician responsible for the management of waste. Therefore, another important obstacle for the HWMP is the lack of trained professionals to implement and monitor the plan, a task that is informally delegated to managers of units or nursing professionals, who do not have any systematic method to help them to carry out this demand.

The nursing sector has a key role in the management of waste, considering that is directly involved in the generation of HW and is often commissioned to the administrative management of healthcare units because of the understanding of the complexity and the organization of these services^4^ ^,^ ^13^. Knowledge about the regulatory aspects concerning the management of HW are essential for the nurses to assess the conditions of the workplace, to train their staff and to alert all other professionals involved as to the inherent risks and the need for proper disposal of different types of HW.

In practice, the presence of these qualified professionals has not happened, which is worrying. In the surgery department of an university hospital in Egypt^14^, it was found that 29% of the nursing staff had a satisfactory notion of waste management. Nationally, a study directed to nurses of the Family Healthcare Strategy Program in the State of Mato Grosso^15^, found that only 20% knew the waste management steps.

It is noteworthy that the requirement of a HWMP deployment is not restricted to large generators, such as hospitals. Primary Healthcare Centers (PHC), which provide basic healthcare services that do not require hospitalization (medical consultations, guidelines, inhalation therapy, bandages, immunizations, application of injectable drugs, collection of samples for laboratory tests, dental treatment and basic medication provision) are also called upon to properly manage their waste, according to the rules in force in the country ^(^ ^3^ ^,^ ^12^.

In December 2015, existed in Brazil 34,951 PHCs in activity^16^. Although each PHC contribute with a small portion of hazardous waste, this generation is significantly magnified when considering all of these units, asymmetrically distributed in the country. Given the precariousness of internal HW management and the lack of collection or adequate treatment services at about 1244 (22.3%) of Brazilian municipalities^17^, it is inferred that a considerable portion is being improperly handled and disposed in the environment.

In this context, in order to support the work of healthcare facilities managers on the issue of management of HW, this paper aims to propose a facilitation tool, to support the diagnosis, formulation and evaluation of HWMP in PHCs, and present the results of the application of this tool in four units of the city of São Paulo. The merit of this instrument is the integration of the interdisciplinary among the subject areas involved and the systematization of current legislation.

## Method

This is a descriptive study, which used as a research method the multiple case study, applied in four PHCs (identified from PHC-A to PHC-D) in the city of São Paulo, Brazil, during the period from February 2011 to February 2012. The selection criteria of the units considered all the PHCs that constituted, at the time, the West Project - a partnership for management between the Faculty of Medicine Foundation of the University of Sao Paulo (USP) and the Municipality of Sao Paulo Administration. The PHC-D was analyzed together with an Emergency Healthcare Unit (called AMA) because they shared some services (pharmacy, administrative areas, kitchen, dressing rooms and shelter for infectious waste) and the outsourced cleaning service.

This study consisted of three stages: i) design of a facilitation tool for diagnosis, formulation and evaluation of HWMP using as indicators: waste generation and performance analysis; ii) application of the tool in four PHCs, resulting in two consecutive diagnoses regarding the management of waste in 2011 and one year after; and iii) comparative evaluation of both diagnoses.

The facilitation tool was formulated from the systematization of the existing legal and regulatory framework in the country, in the state and in the city of São Paulo, and the Brazilian technical standards, resulting in five forms identified from F-I to F-V.

### The indicators used were:

1. Waste generation indicators: in the literature, these indicators are used to assess whether the generating institution adopts measures to reduce waste production and performs the separation into different groups to give the appropriate destination to each group. The indicators selected for this study: *daily amount of total generation and by group* (kg/d and percentage) and two more appropriate indicators to represent the production of waste in services that provide care without hospitalization: generation rate per outpatient assistance* and generation rate per outpatient procedure** performed in critical areas***. To feed the indicators of generation, weighing was performed in each PHC for five consecutive days (one working week of operation), recording the generation of each source sector (critical area and not critical), separately by waste group: A (infectious), D (common: recyclable and non-recyclable) and E (sharps). Radioactive waste (group C) and chemical (group B) were not considered because the former is not generated in the PHC, while the chemicals still present incipient segregation. For infectious materials it was used the sum (A+E) because they are collected and treated jointly in this city. The values jotted down in the form (F-IV) are the average of values found in the five weighing procedures. Additionally, through administrative records in the studied PHCs, it was computed the *daily average of assistances* and the *daily average of procedures* performed in critical areas (recorded in form F-I). The *rate of total waste generation per assistance* is obtained by dividing the daily average of total waste by the daily average of assistances. The *rate of infectious and sharps waste generation* (A+E) results from the ratio of the daily average of waste by the daily average of procedures performed in the sectors that produce such waste (critical areas).

2. Indicator of performance: represented by the scores that resulted from the evaluation of compliance with 142 regulatory requirements contained in Form V (F-V). For completing this form, various techniques were used: survey of secondary data, available documentation (contracts, records of attendance, human resources and routines), interviews with leaders of different sectors, and on-site observation recorded in a diary. During the application of the tool, the information collected by the authors, fed the F-V, which resulted in two diagnoses of managerial and operational situation of the HW of the four PHCs in 2011, and a year later, a period deemed as reasonable for the implementation of the HWMP. 

The evaluation of the HW management performance in each of the PHC under study, resulted from comparing the diagnoses in the two periods considered, before and after the implementation of the plan.

Regarding the ethical aspects, the research project was approved by the Ethics and Research Committees of the Public Health School/USP (OF. COEP 210/10), and of the Municipal Health Department (CEP/SMS 361/10).

## Results 

### Presentation of the facilitation tool

All requirements and applicable legal requirements - for health, environmental and labor issues - were incorporated into the *facilitation tool*. The five forms (F-I to F-V) that make up this instrument are described briefly in the following.

F-I. *Information on the generator facility and responsible*: it involves data on the location, physical and operational structure of the establishment; registration data in the competent agencies; amount of human resources by function; monthly average of assistances and procedures, and liability relating to HW management and security.

F-II. *Characterization of the internal flow of waste by source sector*: it involves the relationship of waste generated separately by groups (A to E) and by source sector. Lends itself to mapping the property in critical and non-critical areas and the determination of the collection flow. These data help in the distribution planning and calculation of the number and capacity of the needed containers, regarding the more efficient segregation and reduction of waste.

F-III. *Description of the external flow and measures to mitigate generation*: identifies the destination of waste indicating the collection companies and receiving units (recycling sorting centers, treatment plants, landfill) of different waste streams. This information becomes important, as the generator is legally co-responsible for external stages of management. In addition, it assists in the decision of minimizing the generation and optimization of costs.

F-IV. *Records of indicators of generation and waste minimization goals*: keeps the record of three indicators, fed with the results obtained by measurements and information gathered in the unit, regarding the number of outpatients assistances and procedures performed during the period: i) *rate of daily average waste generation by group* (A to E) and the respective percentages; ii) *rate of total waste generation per assistance* (kg/assistance); and iii) *rate of infectious and sharps waste generation (A+E) per procedure in critical areas* (kg/procedure).

The use of these indicators enables the evaluation of the institution's performance in relation to minimizing waste both at whole as per sectors, and supports future decision-making. The goals outlined in this form may be checked during the period due for their achievement or at the time of reapplication at a future date.

F-V. *Checklist of regulatory requirements*: this form resulted in 142 regulatory requirements, divided into 3 blocks: B1- documentation required; B2- preventive actions to avoid health and environmental risks and B3- steps of the internal management (segregation, conditioning, internal collection and transport, storage and external collection). 

**Figure 1:**
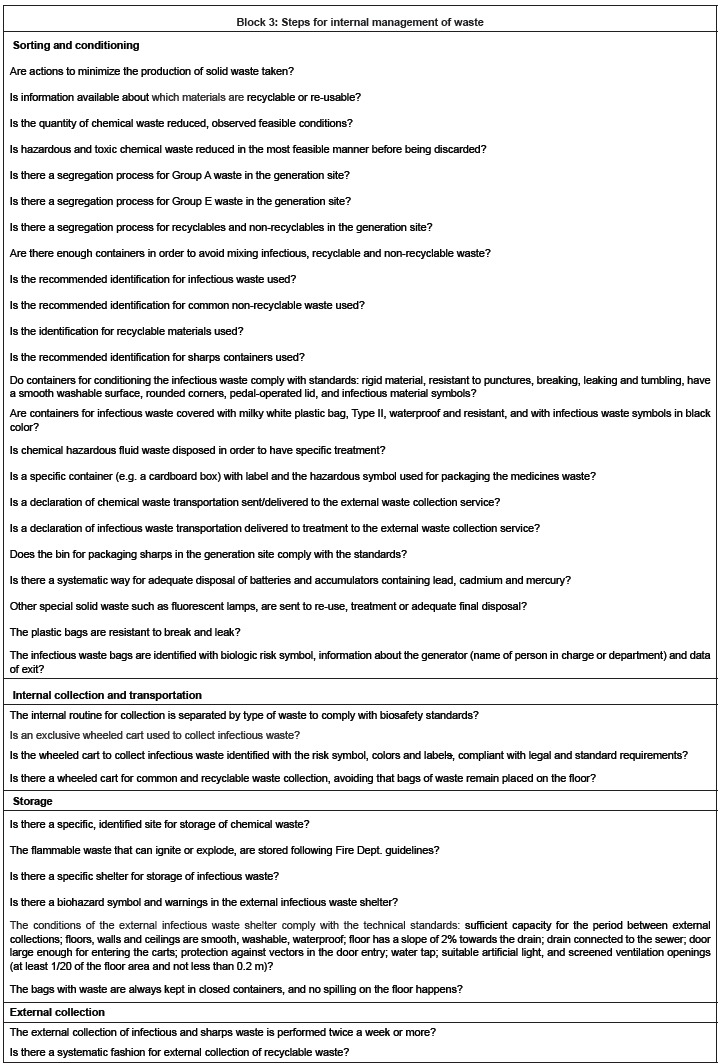
Synthesis of normative requirements of the internal steps in waste management

Answers to the checklist allow assessing whether relevant measures for the management of HW are being adopted. Each requirement may take the following forms: S (Compliant), P (Partially compliant), N (Not compliant), and NA (Not applicable). The un-weighted sum of requirements met, generates a score that reflects the performance of the unit analyzed. The challenge is to reach 100% of positive responses (S). As the documentation is provided, equipment is purchased and used, training programs are developed and operational practices are corrected, the score increases, raising the performance level and stimulating the search for better results. As an illustration, Figure 1 presents main normative requirements composing Block 3, Form V, grouped by steps of management*.

### Presentation of the management situation for HW in the four PHCs under study at two different times

In 2011, all forms of the tool were filled with data at each PHC under study. 

**Figure 2:**
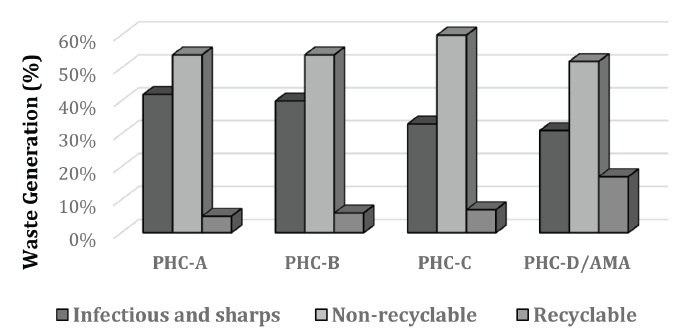
Percentage of waste generation in four Primary Healthcare Centers (PHC), Sao Paulo, SP, Brazil, 2011

Waste quantification (Figure 2) indicated that common waste (non-recyclable) was the highest generated amount (52 to 60%), and few recyclable was separate (5 to 17%), limited to cardboard boxes which were made available to independent waste pickers. The amount of hazardous waste was significant (between 31-42% of total generation). The largest amount of infectious or sharps resulted from the mixture of other wastes (recyclable and not) due to the lack of specific and clearly identified containers.

In 2011, there were significant fluctuations in generation rates in the four PHCs. The PHC-C showed the highest values and PHC-D the smallest (Table 1). Among the four PHCs, the average rate of total waste was 0.09kg/assistance, considering that the average rate of infectious waste was 0.03kg/assistance and 0.09kg/procedure. It is noted that the rate infectious/sharps (A+E) waste generated by procedure exceeds two-to-fourfold the rate per assistance, because procedures are the activities that effectively generate infectious waste.

**Table 1:** Rates of waste generation by Primary Healthcare Center, Sao Paulo, SP, Brazil, 2011

Daily generation	Units	Primary Healthcare Centers			
		PHC-A	PHC-B	PHC-C	PHC-D/AMA
Total (A+E+D)	(kg/assistance)	0,06	0,08	0,17	0,05
Waste (A+E)	(kg/assistance)	0,02	0,03	0,06	0,02
Waste (A+E)	(kg/procedure)	0,08	0,10	0,12	0,05

In the second diagnosis, carried out in 2012, the quantification of waste and the consequent application of the F-IV were affected, considering that management and operating practices to provide the minimization of waste have not been adopted in any of the four PHC. Then, the situation one year after, remained virtually the same as the previous year.

**Figure 3:**
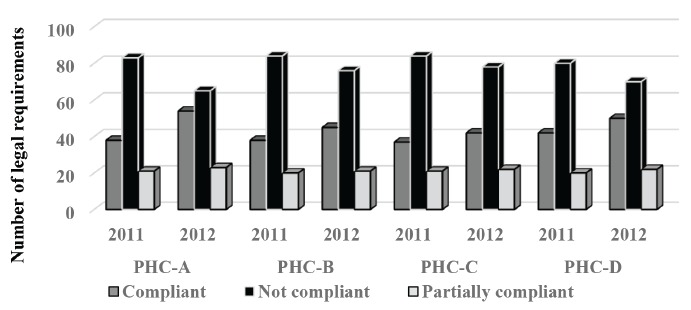
Compliance with legal requirements by Primary Healthcare Centers, Sao Paulo, SP, 2011 and 2012

The application of F-V was feasible in 2011 and 2012. However, the results showed that progress in performance management of HW in the PHC under study, was negligible, considering the 142 items to be met (Figure 3). The progress seen in the PHCs represented: 16 points (PHC-A), 7 points (PHC-B), 5 points (PHC-C) and 8 points (PHC-D).

## Discussion 

Inadequacies in the management of HW and consequent occupational and environmental situations of risk are often highlighted in studies conducted in hospitals in developing countries^5^ ^-^ ^11^. However, it is worth of note that little attention has been directed to the also worrying conditions in non-hospital healthcare facilities such as clinics^3^ ^,^ ^12^ and emergency healthcare units^4^.

In this study, specifically referring to waste on PHCs, management, operation and infrastructure failures have been identified, comparable to those mentioned in the literature. This situation indicates that the legal requirements are not being met, putting at risk the safety of health and cleaning workers as well as users, family caregivers, workers of the external collection, informal waste pickers and even the environment.

In line with previous studies^3^ ^,^ ^12^, it was also verified the lack of job training to practice HW management in the PHCs surveyed. In this case, managers and / or nurses are due to assume this responsibility and individually seek for the improvement of their skills, to enforce the regulations. Moreover, if serious shortcomings are identified, these professionals can even suffer legal / criminal penalties imposed by regulatory agencies of health and environmental competence.

It is noteworthy that, despite the recommendations set out in studies conducted in different countries^5^ ^-^ ^9^ regarding the imperative need to implement plans and institutional policies, the study was unable to find in the literature systematic and standardized methods in order to assist generators facilities to plan and implement these activities. The proposed tool appears to fill this gap and facilitate the performance of this function. Although extensive, it is subdivided into forms that can be applied by one or more staff members, acquainted to these matter. The data thus collected will allow to feed generation and performance indicators.

It is also apparent that the most widely used generation indicator in literature - daily rate per hospital bed (kg of waste/bed.day)^5^ ^-^ ^6^ ^,^ ^8^ ^-^ ^9^ - is generic and suitable for application to establishments that offer more complex healthcare, and requiring the hospitalization of the patient. Fewer studies^3^ ^-^ ^4^ ^,^ ^10^ ^-^ ^12^ present generation rates specific for outpatients or undergoing visits without the need for hospitalization, whether in hospitals, outpatient or emergency units.

In this study, we proposed two more specific indicators to characterize the generation of waste in outpatient units: *generation rate per* assistance *and generation rate per procedure in critical areas*. It is understood that this specificity brings greater reliability to the quantification of waste and is more appropriate for outpatient units. The rate of total waste generation per assistance in the four studied PHCs ranged from 0.05 to 0.17kg (average of 0,11kg). This value appears to be higher than the value found in other PHC in Sao Paulo^12^ (0.03kg/assistance) and nine PHCs in the city of Goiânia^3^ (average of 0,06kg/user). The generation rate found in the PHC-C (0.17kg/assistance), is much larger than the others, indicating the urgent need for measures to minimize the generation of waste.

In the literature is also remarkable the lack of tools to assess the performance of the HW management. Generation and performance indicators as proposed in the present study, lend themselves both to assess progress in the same unit over time, as well as for comparison and ranking of PHCs, at any given time.

In the PHCs under study, the documented way of exposing the nonconformities served as a warning, however it was not enough to motivate their managers to invest in adapting the management of HW. Limiting aspects considered were: the delay in political decision of the PHC responsible for the management, and the lack of human and financial resources to make the needed repairs and improvements.

## Conclusion

The proposed tool seeks to fill the identified gaps enabling the following procedures: Joint visualization of legal requirements dispersed in different regulatory areas involved (health, environment and labor); Application by non-experts on the issue, after brief training; Identification of structural, operational and behavioral failures in the healthcare facility; Easy identification of corrective measures to be implemented after identification of non-conformities; Setting goals and deadlines; Comparison of results in consecutive evaluations, in the same unit, and assessments between different units, evidencing the performance of the HW management; Minimizing the subjectivity of the evaluator; and Achievement of more consistent, reliable and measurable results, for making decisions.

This tool, which organizes all the requirements and legal requirements, may contribute to healthcare management practices, tasks usually attributed to nurses. When fully completed, the tool also has the role of documentary record of the healthcare facility situation at the completion dates.

Because it is an easy-to-handle tool, generating consistent and comparable results, it is recommended to apply this tool in other similar outpatient units, public or private services that have size, type of service and of similar HW characteristics.

It is also recommended that, for dental, veterinary and even hospitals this tool should be adapted to meet the specific needs of these units. For facilities in other municipalities, excluding Sao Paulo should be considered the state and municipal regulations in force. 
